# Progress in Laryngology

**Published:** 1900-09-29

**Authors:** 


### Progress in Laryngology.

(*Continued from page* 423.)

Hopkins,[12] writing upon the use of supra-renal extract, admits its value in the performance of operations, without loss of blood, upon the nose, but at the same time there was danger of secondary hæmorrhage occurring. This was profuse, and came on several hours after the operation. He believes there is more probability of this after the use of supra-renal extract and cocaine than after cocaine alone. Some specialists, alive to this fact, always pack the part after operation as a precautionary measure. Watson found cocaine just as useful as supra-renal extract, the latter only deferring the relaxation.

Lewis Somers[13] points out that atrophic rhinitis being very often secondary to pathological changes in the nasal accessory sinuses, the remedy, whether it be a powder, spray, or solution, does not touch the original seat of the disease. The intolerable offensive odour of this disease may be relieved after cleansing with hydrogen dioxide and an alkaline solution, but Hamm obtains the most excellent results in the treatment of ozœna with citric acid. The nasal chambers should first be cleansed with the solutions mentioned above, and then the powder, finely triturated with an equal quantity of milk sugar, should be blown in with a powder blower. If repeated daily the odour will be dispelled. It is sometimes preferable to commence with a powder containing only 25 per cent. of Ac. citric, and then to gradually increase the strength.

It appears that the percentage of diphtheritic paralysis has increased since the introduction of antitoxin.[14] Paralysis, and the mortality from it, follows less frequently after large doses of the antitoxin, *i.e.*, not less than 4,600 units. The earlier the injections are used in the disease the less likely is paralysis to follow, and if it does occur, it will probably be mild in nature and of short duration. The conclusion is " that the full value of antitoxin is only obtained by using it early and in efficient doses. If this be done, not only is life saved, but tedious complications are prevented, or at least deprived of their dangerous characters."

Marsden gives some interesting cases[15] of diphtheria, in which tracheotomy was performed and antitoxin administered. The cases made steady progress towards recovery, until, in one case, the twenty-third day, and in the other the twentieth, when there was a return of the laryngeal symptoms, and in each case a second tracheotomy was necessary, and the readministration of antitoxin. Marsden concluded that in these cases there was a re-formation of the diphtheritic membrane; and as if to put this conclusion to the test, a third case arose, in which there was a return of the symptoms five weeks after tracheotomy. A re-injection of 2,000 units of antitoxin was given, and after 48 hours the laryngeal symptoms had entirely disappeared. Marsden thinks that the first two cases demonstrate that immunity after an attack of diphtheria has ceased to exist at the expiration of three weeks, if the attack has been terminated by the injection of antitoxin. He considers that it is necessary to repeat the injections of antitoxin every three weeks so long as the exposure to infection lasts.

[12] Med .Rec., May 19, 1900; Brit. Med. Jour., June 16, 1900. [13] Brit. Med. Jour., June 9, 1900. [14] Laryngoscope, Feb., 1900. [15] Med. Chron., May, 1900.

